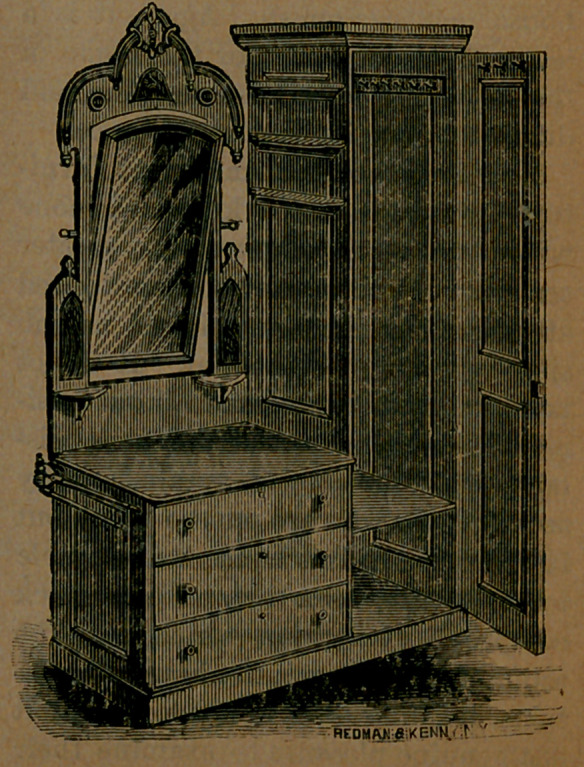# The Economic

**Published:** 1875-02

**Authors:** 


### "THE ECONOMIC."

During a visit to the late Institute Fair in this city, our attention was attracted to a new article of chamber furniture, which struck us as just the thing required in thousands of small rooms, and wherever comfort and neatness are desired, and space is limited. It is a combination, in a single, compact and tasteful piece of furniture, of a wardrobe, washstand, bureau, towel-rack, book-shelves, and looking-glass. It is made in various styles, either of solid walnut or in imitation of walnut or oak, and with marble top, if preferred, and sells for from $18 to $35.

There are several advantages attaching to the use of this new article, which ought to bring it into very general favor. It is economical in every sense, since it occupies only the floor space of a medium-sized bureau, while doing duty for the entire furniture of a bed-chamber, except the bed and chairs. It economises money also, as it satisfactorily supplies the place of six articles costing more than twice as much. There are multitudes of hall bed-rooms and offices, all over the country, in which this elegant and useful combination could be employed to the great saving of space and money. We give below a perfect picture of the article, with its door swinging open. It may be obtained from the manufacturer, Mr. Ambrose E. Barnes, 438 Pearl street, New York City.

## Figures and Tables

**Figure f1:**